# Power and sample size calculations for comparison of two regression lines with heterogeneous variances

**DOI:** 10.1371/journal.pone.0207745

**Published:** 2018-12-17

**Authors:** Gwowen Shieh

**Affiliations:** Department of Management Science, National Chiao Tung University, Hsinchu, Taiwan; University of Adelaide School of Medicine, AUSTRALIA

## Abstract

The existence of interactive effects of a dichotomous treatment variable on the relationship between the continuous predictor and response variables is an essential issue in biological and medical sciences. Also, considerable attention has been devoted to raising awareness of the often-untenable assumption of homogeneous error variance among treatment groups. Although the procedures for detecting interactions between treatment and predictor variables are well documented in the literature, the corresponding problem of power and sample size calculations has received relatively little attention. In order to facilitate interaction design planning, this article describes power and sample size procedures for the extended Welch test of difference between two regression slopes under heterogeneity of variance. Two different formulations are presented to explicate the implications of appropriate reliance on the predictor variables. The simplified method only utilizes the partial information of predictor variances and has the advantages of statistical and computational simplifications. However, extensive numerical investigations showed that it is relatively less accurate than the more profound procedure that accommodates the full distributional features of the predictors. According to the analytic justification and empirical performance, the proposed approach gives reliable solutions to power assessment and sample size determination in the detection of interaction effects. A numerical example involving kidney weigh and body weigh of crossbred diabetic and normal mice is used to illustrate the suggested procedures with flexible allocation schemes. Moreover, the organ and body weights data is incorporated in the accompany SAS and R software programs to illustrate the ease and convenience of the proposed techniques for design planning in interactive research.

## Introduction

Interactive analysis has received fairly extensive attention in biological and medical sciences. The occurrence of interaction implies that the influence of a predictor variable on the response variable varies as a function of the level of a treatment variable. In general, the existence and magnitude of interaction effects can be measured and appraised by the statistical methods for analyzing the product terms of treatment and predictor variables. Alternatively, the procedure for evaluating the interaction effects between treatment and predictor variables is methodologically identical to that for testing the equality of regression slope coefficients in two or more regression lines. The common testing procedures of multiple linear regression described by Fleiss [1], Kleinbaum et al. [2], and Kutner et al. [3] can be readily applied for the detection of different forms of interaction effects. Among various pedagogical development and exposition of interaction studies, particular emphasis has been paid to the fundamental attributes of two treatment groups, such as Hewett and Lababidi [4], Hill and Padmanabhan [5], Spurrier, Hewett, and Lababidi [6], and Tsutakawa and Hewett [7], among others.

The traditional multiple linear regression adopts the fundamental assumptions of independence, normality, and constant variance. Accordingly, homogeneity of error variance is one of the essential assumptions for the appropriate use of common test procedures. The detrimental effects of heterogeneous error variance on the regular test procedures have been investigated in Alexander and DeShon [8], DeShon and Alexander [9], Dretzke, Levin, and Serlin [10], Overton [11], and Shieh [12]. The numerical findings indicated that the resulting Type I error rate and power levels may be considerably affected when group sample sizes are equal, and severely distorted when group sample sizes are unequal. Therefore, the traditional tests of interaction effects are not robust to the heterogeneity of error variance for evaluating regression slope differences across groups. It is emphasized in Dretzke et al. [10] and Shieh [12] that the importance of choosing a proper test procedure for two regression slope differences when group error variances are unequal. Moreover, under the prevalent scenario of variance heterogeneity, a theory-based approach to power calculation for assessing the existence of treatment by predictor interactions remains unavailable.

To enhance the adoption of appropriate techniques for statistical inference and research design, this article aims to present power and sample size procedures for the well-recognized Welch [13] test of parallelism of two regression lines with heterogeneous variances. Accordingly, the suggested techniques are direct extensions of those considered in Shieh [14] under a homogeneous variance setting, just as the Welch [13] procedure to the traditional two-sample *t* test for mean comparisons. Four important aspects of this study should be pointed out. First, the primitive Welch solution to the Behrens-Fisher problem for comparing the means of two groups under heterogeneity of variance has been widely discussed in the literature (Kim and Cohen [15]). Although there exist several viable alternatives to mitigate the impact of violating the assumption of homogeneous error variance, the accurate performance and computational ease demonstrated in Dretzke et al. [10] and Shieh [12] ensure that the extended Welch procedure for regression slope equality can be recommended. Second, the actual values of predictors cannot be known in advance before conducting a research study just as the primary responses. In order to account for the stochastic nature of predictors, two different general or unconditional distributions of the extended Welch statistic are described to obtain feasible power functions and sample size formulas. Third, comprehensive numerical assessments were conducted to illustrate the discrepancy between two potential approaches under a wide range of model configurations. The empirical findings revealed that the more elaborate procedure generally outperforms the alternative simplified method for correctly accommodating the predictor features. Fourth, to ease the computational demands of the proposed power and sample size calculations, both SAS and R computer algorithms are presented. The program codes are computationally efficient and will give accurate outputs when all the necessary information is properly specified. With the most plausible model configurations, the recommended techniques are practically useful for power analysis and sample size determination in planning interaction studies.

## Methods

Consider the two simple linear regression models with unknown and possibly unequal coefficient parameters and error variances:

$$Y_{1j}=\beta_{01}+\beta_{11}X_{1j}+\varepsilon_{1j}\;and\;{Y_{2k}=\beta }_{02}+\beta_{12}X_{2k}+\varepsilon_{2k},$$ (1)

where *ε*_1*j*_ and *ε*_1*k*_ are *iid* ${N(0,\sigma }_{1}^{2})$ and ${N(0,\sigma }_{2}^{2})$) random variables, respectively, *j* = 1, …, *N*_1_, and *k* = 1, …, *N*_2_. Note that Shieh [14] focused on the scenario with homogeneous error variance $\sigma_{1}^{2}=\sigma_{2}^{2}$. Moreover, the regression model with unequal slopes in Eq 1 can be formulated as an interactive model using a dichotomous treatment variable *M*:

$${Y_{i}=\beta }_{02}+\beta_{0D}M_{i}+\beta_{12}X_{i}+\beta_{1D}M_{i}X_{i}+\varepsilon_{i},\;i=1,\ldots ,{N_{T},N_{T}=N_{1}+N}_{2},$$ (2)

$${where\;\beta }_{0D}=\beta_{01}-\beta_{02},\;\beta_{1D}=\beta_{11}-\beta_{12};$$

$$Y_{i}=Y_{1j},\;X_{i}=X_{1j},\;\varepsilon_{i}=\varepsilon_{1j},\;and\;M_{i}={1\;if\;i=j,j=1,\ldots ,N}_{1};$$

$$Y_{i}=Y_{2k},\;X_{i}=X_{2k},\;\varepsilon_{i}=\varepsilon_{2k},\;and\;M_{i}={0\;if\;i=N_{1}+k,k=1,\ldots ,N}_{2}.$$

Evidently, the slope difference between the two regression lines *β*_1*D*_ = *β*_11_−*β*_12_ is also the coefficient associated with the cross-product term between the predictor and treatment variables. The existence and magnitude of *β*_1*D*_ indicates the influences of a dichotomous treatment indicator on the direction and/or the strength of the relationship between the response and predictor variables.

For the purpose of detecting the interaction effect, the problem is naturally concerned with the hypothesis

$$H_{0}:\beta_{1D}=0\;versus\;H_{1}:\beta_{1D}\neq 0$$ (3)

and the distributional properties of the corresponding least squares estimators of slope coefficients ${\hat{\beta }}_{11}$ and ${\hat{\beta }}_{12}$. Under the model formulation in Eq 1, the standard results show that ${\hat{\beta }}_{11}∼N(\beta_{11},\sigma_{1}^{2}/{SSX}_{1})$ and ${\hat{\beta }}_{12}∼N(\beta_{12},\sigma_{2}^{2}/{SSX}_{2})$ where ${SSX}_{1}=\sum_{j=1}^{N_{1}}{\left(X_{1j}-{\bar{X}}_{1}\right)}^{2},\;{SSX}_{2}=\sum_{k=1}^{N_{2}}{\left(X_{2k}-{\bar{X}}_{2}\right)}^{2}$, and ${\bar{X}}_{1}$ and ${\bar{X}}_{2}$ are the respective sample means of the *X*_1*j*_ and *X*_2*k*_ observations. These results lead to ${\hat{\beta }}_{1D}∼N(\beta_{1D},\sigma_{1}^{2}/{SSX}_{1}+\sigma_{2}^{2}/{SSX}_{2})$ where ${\hat{\beta }}_{1D}={\hat{\beta }}_{11}-{\hat{\beta }}_{12}$. Also, ${\hat{\sigma }}_{1}^{2}={SSE}_{1}/c_{1}$ and ${\hat{\sigma }}_{2}^{2}={SSE}_{2}/c_{2}$ are the usual unbiased estimators of $\sigma_{1}^{2}$ and $\sigma_{2}^{2}$ where *SSE*_1_ and *SSE*_2_ are the separate group error sum of squares, *c*_1_ = *N*_1_−2, and *c*_2_ = *N*_2_−2. Moreover, $V_{1}={c_{1}\hat{\sigma }}_{1}^{2}/\sigma_{1}^{2}∼\chi^{2}(c_{1})$ and $V_{2}={c_{2}\hat{\sigma }}_{2}^{2}/\sigma_{2}^{2}∼\chi^{2}(c_{2})$, where *χ*^2^(*c*_1_) and *χ*^2^(*c*_2_) are chi-square distribution with *c*_1_ and *c*_2_ degrees of freedom, respectively.

To detect the non-parallelism of two regression lines or the interactive effects of a dichotomous treatment variable between the predictor and response variables in terms of H_0_: *β*_1*D*_ = 0 versus H_1_: *β*_1*D*_ ≠ 0, Welch [13] proposed an extension of the well-known approximate degrees of freedom solution to the Behren-Fisher problem of comparing the difference between two means. Accordingly, the extended Welch statistic has the form:

$$T^{*}=\frac{{\hat{\beta }}_{1D}}{{\left({\hat{\sigma }}_{1}^{2}/{SSX}_{1}+{\hat{\sigma }}_{2}^{2}/{SSX}_{2}\right)}^{1/2}}.$$ (4)

Under the null hypothesis H_0_: *β*_1*D*_ = 0, Welch [13] considered the approximate distribution for T*:

$$T^{*}\dot{∼}t(\hat{\nu })$$ (5)

where $t(\hat{\nu })$ is a *t* distribution with degrees of freedom $\hat{\nu }$ and

$$1/\hat{\nu }=\frac{1}{c_{1}}{\left\{\frac{{\hat{\sigma }}_{1}^{2}/{SSX}_{1}}{{\hat{\sigma }}_{1}^{2}/{SSX}_{1}+{\hat{\sigma }}_{2}^{2}/{SSX}_{2}}\right\}}^{2}+\frac{1}{c_{2}}{\left\{\frac{{\hat{\sigma }}_{2}^{2}/{SSX}_{2}}{{\hat{\sigma }}_{1}^{2}/{SSX}_{1}+{\hat{\sigma }}_{2}^{2}/{SSX}_{2}}\right\}}^{2}.$$

The null hypothesis is rejected at the significance level *α* if

$$|T^{*}|>t_{\hat{\nu },\alpha /2},$$ (6)

where $t_{\hat{\nu },\alpha /2}$ is the 100(1−*α*/2) percentile of the *t* distribution $t(\hat{\nu })$ with degrees of freedom $\hat{\nu }$.

The statistical inferences about the interactive effect are based on the conditional distribution of the continuous outcomes {*X*_1*j*_, *j* = 1, …, *N*_1_} and {*X*_2*k*_, *k* = 1, …, *N*_2_}. Consequently, the particular results would depend on the observed values of the predictor variables. At the design stage, however, the actual values of both the response and predictor variables cannot be determined. Hence, it is more appropriate to consider the random or unconditional framework as demonstrated in Binkley and Abbot [16], Cramer and Appelbaum [17], Gatsonis and Sampson [18], and Sampson [19]. Although the unconditional properties of the test procedure are relatively more involved, the associated hypothesis testing and parameter estimation remain the same under both conditional and unconditional modeling settings. The fundamental distinction between the two modeling scenarios is essential when performing power and sample size calculations. It is of theoretical importance to recognize the random characteristic of the predictor variables and to appraise the unconditional property of the test statistic over the possible values of the predictors.

In order to explicate the critical notion of accommodating the distributional properties of the predictor variables, two different power functions are presented in Appendix. The simplified *t* method only utilizes the partial information of predictor variances. The associated power function Ψ_*ST*_ defined in Eq A3 has the advantages of statistical and computational simplifications. On the other hand, the mixed *t* approach considers the improved power function Ψ_*MT*_ given in Eq A6 and accommodates the full distributional features of the predictors. Despite the close resemblance between the two power formulas, the corresponding behavior may differ substantially for finite samples. In practice, a research study requires adequate statistical power and sufficient sample size to detect scientifically credible effects. For the purpose of planning interaction design, the prescribed two power formulas can be employed to determine the sample sizes *N*_1_ and *N*_2_ needed to attain the specified power through a simple iterative search for the chosen significance level and model configurations. Accordingly, it is of practical interest and theoretical importance to examine the underlying characteristics of the two approaches in power and sample size calculations.

## Simulation study

Due to the complexity of the power and sample size problem, a complete theoretical evaluation of the associated issues is not feasible. To clarify the similarities and differences of the prescribed two different distributions for the extended Welch statistic, extensive numerical appraisals were conducted for their performance in power and sample size calculations. The investigation was carried out in two steps. The first step involved sample size determinations across a wide range of manipulated model configurations. In the second step, a Monte Carlo simulation study was performed to compare the accuracy of the alternative sample size formulas through power assessments under the design characteristics specified in the first step.

### Sample size determinations

The determination of necessary sample size for testing regression slope differences requires detailed specifications of the magnitudes of slope coefficients, error variances, predictor variances, nominal power, and significance level. Notably, the impact of each of these critical factors on sample size calculations not only differs but also varies with the coexisting magnitudes of other components. To provide a systematic evaluation, the empirical examinations are administered by fixing all but one of the following critical attributes and by varying a single component in the appraisal: (1) slope differences, (2) sample size ratios, (3) predictor variances, and (4) error variances. Specifically, the magnitudes of slope coefficients are chosen as *β*_11_ = {0.50, 0.75, 1.00} and *β*_12_ = 0 to represent small, medium, and large slope differences with *β*_1*D*_ = {0.50, 0.75, 1.00}. Both balanced and unbalanced designs are considered with sample size ratio *r* = *N*_2_/*N*_1_ = 1 and 3. The predictor variances are assumed to have three different structures: $\left\{\tau_{1}^{2},\tau_{2}^{2}\right\}$ = {1, 1}, {1, 4}, and {4, 1}. Moreover, a total of five patterns of error variances are evaluated: $\left\{\sigma_{1}^{2},\sigma_{2}^{2}\right\}$ = {1, 1}, {1, 2}, {1, 4}, {2, 1}, and {4, 1}. The diverse settings not only include both balanced and unbalanced schemes, but also generate direct- and inverse-pairing with variance component patterns. Note that the empirical investigation involves a total of 90 combined conditions. It should be emphasized that these model configurations are designated to represent the possible extent of characteristics in practical study without unrealistic or excessive settings. In fact, similar setups were considered in Overton [11] and Shieh [12]. Throughout these numerical comparisons, the significance level and nominal power are set as *α* = 0.05 and 1−*β* = 0.80, respectively.

With the aforementioned specifications, the necessary sample sizes can be computed by the supplemental SAS/IML (SAS Institute [20]) or R (R Development Core Team [21]) programs for the simplified *t* and mixed *t* methods with the power functions defined in Eqs A3 and A6, respectively. The resulting sample sizes {*N*_1_, *N*_2_} associated with the slope difference *β*_1*D*_ = {0.05, 0.75, 1.00} in combination with sample size ratio *r* = {1, 3} are summarized in Tables 1–6, respectively. Accordingly, there are 15 combined cases of $\left\{\tau_{1}^{2},\tau_{2}^{2}\right\}$ and $\left\{\sigma_{1}^{2},\sigma_{2}^{2}\right\}$ in each table. For ease of illustration, the total sample size *N*_*T*_ and the working effect size δ* given in Eq A4 are also presented. As expected, the computed sample sizes increase with smaller magnitude of slope difference *β*_1*D*_ or effect size δ*. Also, the necessary sample sizes associated with larger error variances $\left\{\sigma_{1}^{2},\sigma_{2}^{2}\right\}$ are consistently larger than those of the smaller error variances when all other factors are held constant. However, the situation is reversed in terms of the predictor variances. In other words, the scenarios with larger $\left\{\tau_{1}^{2},\tau_{2}^{2}\right\}$ incurred smaller sample sizes than those with smaller predictor variances when all other characteristics remain the same.

**Table 1:** Computed sample size, estimated power, and simulated power when the slope difference *β*_1*D*_ = 0.50, sample size ratio *r* = 1, Type I error *α* = 0.05, and nominal power 1−*β* = 0.80.

				Simplified *t* method						Mixed *t* method					
Case	$\tau_{1}^{2}/\tau_{2}^{2}$	$\sigma_{1}^{2}/\sigma_{2}^{2}$	δ*	*N*_1_	*N*_2_	*N*_*T*_	Estimated power	Simulated power	Error	*N*_1_	*N*_2_	*N*_*T*_	Estimated power	Simulated power	Error
1	1/1	1/1	0.0218	65	65	130	0.8015	0.7954	0.0061	67	67	134	0.8025	0.7993	0.0032
2	1/1	1/2	0.0146	97	97	194	0.8030	0.7998	0.0032	99	99	198	0.8038	0.7992	0.0046
3	1/1	1/4	0.0088	160	160	320	0.8018	0.7983	0.0035	162	162	324	0.8023	0.7967	0.0056
4	1/1	2/1	0.0146	97	97	194	0.8030	0.8003	0.0027	99	99	198	0.8038	0.8042	–0.0004
5	1/1	4/1	0.0088	160	160	320	0.8018	0.7956	0.0062	162	162	324	0.8023	0.7983	0.0040
6	1/4	1/1	0.0341	42	42	84	0.8042	0.7872	0.0170	44	44	88	0.8067	0.8075	–0.0008
7	1/4	1/2	0.0286	50	50	100	0.8067	0.7927	0.0140	51	51	102	0.8003	0.8030	–0.0027
8	1/4	1/4	0.0218	65	65	130	0.8015	0.7946	0.0069	67	67	134	0.8025	0.7996	0.0029
9	1/4	2/1	0.0192	74	74	148	0.8044	0.7957	0.0087	75	75	150	0.8006	0.7944	0.0062
10	1/4	4/1	0.0103	137	137	274	0.8025	0.8015	0.0010	138	138	276	0.8005	0.7975	0.0030
11	4/1	1/1	0.0341	42	42	84	0.8042	0.7908	0.0134	44	44	88	0.8067	0.8072	–0.0005
12	4/1	1/2	0.0192	74	74	148	0.8044	0.7969	0.0075	75	75	150	0.8006	0.8031	–0.0025
13	4/1	1/4	0.0103	137	137	274	0.8025	0.7984	0.0041	138	138	276	0.8005	0.8033	–0.0028
14	4/1	2/1	0.0286	50	50	100	0.8067	0.7892	0.0175	51	51	102	0.8003	0.8024	–0.0021
15	4/1	4/1	0.0218	65	65	130	0.8015	0.7918	0.0097	67	67	134	0.8025	0.8058	–0.0033

Note: $\left\{\tau_{1}^{2},\tau_{2}^{2}\right\}$ and $\left\{\sigma_{1}^{2},\sigma_{2}^{2}\right\}$ are the predictor variances and error variances of the two groups, and δ* is the standardized slope difference.

**Table 2:** Computed sample size, estimated power, and simulated power when the slope difference *β*_1*D*_ = 0.50, sample size ratio *r* = 3, Type I error *α* = 0.05, and nominal power 1−*β* = 0.80.

				Simplified *t* method						Mixed *t* method					
Case	$\tau_{1}^{2}/\tau_{2}^{2}$	$\sigma_{1}^{2}/\sigma_{2}^{2}$	δ*	*N*_1_	*N*_2_	*N*_*T*_	Estimated power	Simulated power	Error	*N*_1_	*N*_2_	*N*_*T*_	Estimated power	Simulated power	Error
1	1/1	1/1	0.0162	44	132	176	0.8014	0.7971	0.0043	46	138	184	0.8063	0.8080	–0.0017
2	1/1	1/2	0.0131	54	162	216	0.8009	0.7937	0.0072	56	168	224	0.8058	0.8015	0.0043
3	1/1	1/4	0.0094	75	225	300	0.8029	0.7906	0.0123	76	228	304	0.8021	0.7988	0.0033
4	1/1	2/1	0.0093	76	228	304	0.8021	0.7956	0.0065	78	234	312	0.8044	0.8025	0.0019
5	1/1	4/1	0.0051	139	417	556	0.8010	0.7959	0.0051	141	423	564	0.8021	0.8056	–0.0035
6	1/4	1/1	0.0195	37	111	148	0.8040	0.7892	0.0148	39	117	156	0.8088	0.8098	–0.0010
7	1/4	1/2	0.0181	40	120	160	0.8104	0.7904	0.0200	41	123	164	0.8050	0.7966	0.0084
8	1/4	1/4	0.0162	44	132	176	0.8014	0.7901	0.0113	46	138	184	0.8063	0.8111	–0.0048
9	1/4	2/1	0.0104	69	207	276	0.8049	0.7944	0.0105	70	210	280	0.8013	0.7951	0.0062
10	1/4	4/1	0.0054	132	396	528	0.8029	0.7972	0.0057	133	399	532	0.8010	0.8047	–0.0037
11	4/1	1/1	0.0360	20	60	80	0.8097	0.7845	0.0252	21	63	84	0.8061	0.7980	0.0081
12	4/1	1/2	0.0236	30	90	120	0.8029	0.7911	0.0118	31	93	124	0.8033	0.8029	0.0004
13	4/1	1/4	0.0139	51	153	204	0.8027	0.7926	0.0101	52	156	208	0.8043	0.8097	–0.0054
14	4/1	2/1	0.0255	28	84	112	0.8039	0.7872	0.0167	30	90	120	0.8132	0.8095	0.0037
15	4/1	4/1	0.0162	44	132	176	0.8014	0.7951	0.0063	46	138	184	0.8063	0.8049	0.0014

Note: $\left\{\tau_{1}^{2},\tau_{2}^{2}\right\}$ and $\left\{\sigma_{1}^{2},\sigma_{2}^{2}\right\}$ are the predictor variances and error variances of the two groups, and δ* is the standardized slope difference.

**Table 3:** Computed sample size, estimated power, and simulated power when the slope difference *β*_1*D*_ = 0.75, sample size ratio *r* = 1, Type I error *α* = 0.05, and nominal power 1−*β* = 0.80.

				Simplified *t* method						Mixed *t* method					
Case	$\tau_{1}^{2}/\tau_{2}^{2}$	$\sigma_{1}^{2}/\sigma_{2}^{2}$	δ*	*N*_1_	*N*_2_	*N*_*T*_	Estimated power	Simulated power	Error	*N*_1_	*N*_2_	*N*_*T*_	Estimated power	Simulated power	Error
1	1/1	1/1	0.0476	30	30	60	0.8014	0.7767	0.0247	32	32	64	0.8041	0.8055	–0.0014
2	1/1	1/2	0.0323	44	44	88	0.8005	0.7802	0.0203	46	46	92	0.8024	0.7989	0.0035
3	1/1	1/4	0.0195	73	73	146	0.8051	0.7933	0.0118	74	74	148	0.8009	0.8065	–0.0056
4	1/1	2/1	0.0323	44	44	88	0.8005	0.7809	0.0196	46	46	92	0.8024	0.8034	–0.0010
5	1/1	4/1	0.0195	73	73	146	0.8051	0.7884	0.0167	74	74	148	0.8009	0.7973	0.0036
6	1/4	1/1	0.0731	20	20	40	0.8042	0.7683	0.0359	22	22	44	0.8112	0.8103	0.0009
7	1/4	1/2	0.0612	24	24	48	0.8173	0.7885	0.0288	25	25	50	0.8039	0.7896	0.0143
8	1/4	1/4	0.0476	30	30	60	0.8014	0.7740	0.0274	32	32	64	0.8041	0.8039	0.0002
9	1/4	2/1	0.0422	34	34	68	0.8003	0.7827	0.0176	36	36	72	0.8045	0.8029	0.0016
10	1/4	4/1	0.0227	63	63	126	0.8063	0.8017	0.0046	64	64	128	0.8020	0.7996	0.0024
11	4/1	1/1	0.0731	20	20	40	0.8042	0.7664	0.0378	22	22	44	0.8112	0.8180	–0.0068
12	4/1	1/2	0.0422	34	34	68	0.8003	0.7837	0.0166	36	36	72	0.8045	0.8055	–0.0010
13	4/1	1/4	0.0227	63	63	126	0.8063	0.7955	0.0108	64	64	128	0.8020	0.7965	0.0055
14	4/1	2/1	0.0612	24	24	48	0.8173	0.7826	0.0347	25	25	50	0.8039	0.8026	0.0013
15	4/1	4/1	0.0476	30	30	60	0.8014	0.7717	0.0297	32	32	64	0.8041	0.8014	0.0027

Note: $\left\{\tau_{1}^{2},\tau_{2}^{2}\right\}$ and $\left\{\sigma_{1}^{2},\sigma_{2}^{2}\right\}$ are the predictor variances and error variances of the two groups, and δ* is the standardized slope difference.

**Table 4:** Computed sample size, estimated power, and simulated power when the slope difference *β*_1*D*_ = 0.75, sample size ratio *r* = 3, Type I error *α* = 0.05, and nominal power 1−*β* = 0.80.

				Simplified *t* method						Mixed *t* method					
Case	$\tau_{1}^{2}/\tau_{2}^{2}$	$\sigma_{1}^{2}/\sigma_{2}^{2}$	δ*	*N*_1_	*N*_2_	*N*_*T*_	Estimated power	Simulated power	Error	*N*_1_	*N*_2_	*N*_*T*_	Estimated power	Simulated power	Error
1	1/1	1/1	0.0347	21	63	84	0.8075	0.7740	0.0335	23	69	92	0.8181	0.8151	0.0030
2	1/1	1/2	0.0286	25	75	100	0.8028	0.7818	0.0210	27	81	108	0.8132	0.8152	–0.0020
3	1/1	1/4	0.0209	34	102	136	0.8028	0.7875	0.0153	35	105	140	0.8009	0.8002	0.0007
4	1/1	2/1	0.0205	35	105	140	0.8007	0.7849	0.0158	37	111	148	0.8064	0.8073	–0.0009
5	1/1	4/1	0.0112	64	192	256	0.8059	0.7942	0.0117	65	195	260	0.8002	0.8098	–0.0076
6	1/4	1/1	0.0413	18	54	130	0.8049	0.7685	0.0364	20	60	80	0.8169	0.8168	0.0001
7	1/4	1/2	0.0389	19	57	76	0.8070	0.7735	0.0335	21	63	84	0.8182	0.8188	–0.0006
8	1/4	1/4	0.0347	21	63	84	0.8075	0.7855	0.0220	23	69	92	0.8181	0.8161	0.0020
9	1/4	2/1	0.0226	32	64	96	0.8017	0.7835	0.0182	34	102	136	0.8074	0.8067	0.0007
10	1/4	4/1	0.0119	60	180	240	0.8011	0.7944	0.0067	62	186	248	0.8037	0.8073	–0.0036
11	4/1	1/1	0.0751	10	30	40	0.8266	0.7794	0.0472	11	33	44	0.8175	0.8199	–0.0024
12	4/1	1/2	0.0514	14	42	56	0.8056	0.7757	0.0299	15	45	60	0.8057	0.8102	–0.0045
13	4/1	1/4	0.0301	24	72	96	0.8164	0.8051	0.0113	24	72	96	0.8028	0.8043	–0.0015
14	4/1	2/1	0.0534	14	42	56	0.8238	0.7877	0.0361	15	45	60	0.8134	0.8113	0.0021
15	4/1	4/1	0.0347	21	63	84	0.8075	0.7759	0.0316	23	69	92	0.8181	0.8125	0.0056

Note: $\left\{\tau_{1}^{2},\tau_{2}^{2}\right\}$ and $\left\{\sigma_{1}^{2},\sigma_{2}^{2}\right\}$ are the predictor variances and error variances of the two groups, and δ* is the standardized slope difference.

**Table 5:** Computed sample size, estimated power, and simulated power when the slope difference *β*_1*D*_ = 1.00, sample size ratio *r* = 1, Type I error *α* = 0.05, and nominal power 1−*β* = 0.80.

				Simplified *t* method						Mixed *t* method					
Case	$\tau_{1}^{2}/\tau_{2}^{2}$	$\sigma_{1}^{2}/\sigma_{2}^{2}$	δ*	*N*_1_	*N*_2_	*N*_*T*_	Estimated power	Simulated power	Error	*N*_1_	*N*_2_	*N*_*T*_	Estimated power	Simulated power	Error
1	1/1	1/1	0.0810	18	18	36	0.8070	0.7634	0.0436	20	20	40	0.8120	0.8097	0.0023
2	1/1	1/2	0.0555	26	26	52	0.8057	0.7860	0.0197	28	28	56	0.8093	0.8054	0.0039
3	1/1	1/4	0.0341	42	42	84	0.8042	0.7899	0.0143	44	44	88	0.8067	0.8108	–0.0041
4	1/1	2/1	0.0555	26	26	52	0.8057	0.7777	0.0280	28	28	56	0.8093	0.8069	0.0024
5	1/1	4/1	0.0341	42	42	84	0.8042	0.7974	0.0068	44	44	88	0.8067	0.8016	0.0051
6	1/4	1/1	0.1192	13	13	26	0.8290	0.7725	0.0565	14	14	28	0.8082	0.8131	–0.0049
7	1/4	1/2	0.1051	14	14	28	0.8026	0.7510	0.0516	16	16	32	0.8119	0.8159	–0.0040
8	1/4	1/4	0.0810	18	18	36	0.8070	0.7677	0.0393	20	20	40	0.8120	0.8105	0.0015
9	1/4	2/1	0.0710	21	21	42	0.8156	0.7858	0.0298	22	22	44	0.8034	0.8026	0.0008
10	1/4	4/1	0.0393	37	37	74	0.8102	0.7897	0.0205	38	38	76	0.8032	0.8070	–0.0038
11	4/1	1/1	0.1192	13	13	26	0.8090	0.7700	0.0590	14	14	28	0.8082	0.8130	–0.0048
12	4/1	1/2	0.0710	21	21	42	0.8156	0.7873	0.0283	22	22	44	0.8034	0.8001	0.0033
13	4/1	1/4	0.0393	37	37	74	0.8102	0.7924	0.0178	38	38	76	0.8032	0.7985	0.0047
14	4/1	2/1	0.1051	14	14	28	0.8026	0.7514	0.0512	16	16	32	0.8119	0.8127	–0.0008
15	4/1	4/1	0.0810	18	18	36	0.8070	0.7553	0.0517	20	20	40	0.8120	0.8062	0.0058

Note: $\left\{\tau_{1}^{2},\tau_{2}^{2}\right\}$ and $\left\{\sigma_{1}^{2},\sigma_{2}^{2}\right\}$ are the predictor variances and error variances of the two groups, and δ* is the standardized slope difference.

**Table 6:** Computed sample size, estimated power, and simulated power when the slope difference *β*_1*D*_ = 1.00, sample size ratio *r* = 3, Type I error *α* = 0.05, and nominal power 1−*β* = 0.80.

				Simplified *t* method						Mixed *t* method					
Case	$\tau_{1}^{2}/\tau_{2}^{2}$	$\sigma_{1}^{2}/\sigma_{2}^{2}$	δ*	*N*_1_	*N*_2_	*N*_*T*_	Estimated power	Simulated power	Error	*N*_1_	*N*_2_	*N*_*T*_	Estimated power	Simulated power	Error
1	1/1	1/1	0.0581	13	39	52	0.8160	0.7717	0.0443	14	42	56	0.8035	0.8031	0.0004
2	1/1	1/2	0.0487	15	45	60	0.8088	0.7668	0.0420	17	51	68	0.8261	0.8307	–0.0046
3	1/1	1/4	0.0360	20	60	80	0.8097	0.7835	0.0262	21	63	84	0.8061	0.8089	–0.0028
4	1/1	2/1	0.0349	21	63	84	0.8057	0.7786	0.0271	23	69	92	0.8157	0.8155	0.0002
5	1/1	4/1	0.0195	37	111	148	0.8040	0.7899	0.0141	39	117	156	0.8088	0.8055	–0.0030
6	1/4	1/1	0.0665	12	36	48	0.8317	0.7763	0.0554	13	39	52	0.8158	0.8152	0.0006
7	1/4	1/2	0.0642	12	36	48	0.8145	0.7576	0.0569	13	39	52	0.8011	0.7942	0.0069
8	1/4	1/4	0.0581	13	39	52	0.8160	0.7674	0.0486	14	42	56	0.8035	0.8019	0.0016
9	1/4	2/1	0.0378	20	60	80	0.8186	0.7854	0.0332	21	63	84	0.8075	0.8060	0.0015
10	1/4	4/1	0.0206	35	105	140	0.8009	0.7835	0.0174	37	111	148	0.8059	0.8054	0.0005
11	4/1	1/1	0.1263	6	18	24	0.8050	0.6954	0.1096	8	24	32	0.8578	0.8551	0.0027
12	4/1	1/2	0.0845	9	27	36	0.8378	0.7780	0.0598	10	30	40	0.8347	0.8339	0.0008
13	4/1	1/4	0.0523	14	42	56	0.8187	0.7968	0.0219	15	45	60	0.8232	0.8243	–0.0011
14	4/1	2/1	0.0874	9	27	36	0.8413	0.7685	0.0728	10	30	40	0.8246	0.8219	0.0027
15	4/1	4/1	0.0581	13	39	52	0.8160	0.7658	0.0502	14	42	56	0.8035	0.8055	–0.0020

Note: $\left\{\tau_{1}^{2},\tau_{2}^{2}\right\}$ and $\left\{\sigma_{1}^{2},\sigma_{2}^{2}\right\}$ are the predictor variances and error variances of the two groups, and δ* is the standardized slope difference.

Also, the unbalanced designs often demand larger sample size to attain the same nominal power than the balanced designs. But there are some exceptions, for example, Cases 3, 11, 12, and 13 in Tables 1 and 2 signify the notable situations. Specifically, the total sample size *N*_*T*_ = 320 and 324 are slightly larger than those of *N*_*T*_ = 300 and 304 for the two methods associated with Case 3 in Tables 1 and 2, respectively. More importantly, the sample sizes of the simplified *t* method are relatively smaller than those of the mixed *t* approach. The only one situation with the same sample sizes is associated with {*N*_1_, *N*_2_} = {24, 72} of Case 13 in Table 4 when *β*_1*D*_ = 0.75, $\left\{\tau_{1}^{2},\tau_{2}^{2}\right\}$ = {4, 1}, and $\left\{\sigma_{1}^{2},\sigma_{2}^{2}\right\}$ = {1, 4}. However, with the two different power functions Ψ_*ST*_ and Ψ_*MT*_, the corresponding estimated powers are 0.8164 and 0.8028, respectively. Note that if problems or deficiencies were to be seen with the alternative power and sample size procedures, they would be most apparent with small sample sizes. Thus, some of these sample sizes are designated to be smaller than those in related studies. In order to evaluate the accuracy of the two power functions, the estimated power or computed power are also listed. Because of the underlying metric of integer sample sizes, the attained values are slightly larger than the nominal level for both procedures.

### Simulation results

To investigate the accuracy of sample size determinations, Monte Carlo simulations were performed in the second stage. With the designated parameter configurations and computed sample sizes, estimates of the true power were computed via Monte Carlo simulation of 10,000 independent data sets for both approaches. For each replicate, {*N*_1_, *N*_2_} predictor values were generated from the selected normal distributions. The resulting values of predictor variables in turn determined the mean responses for generating {*N*_1_, *N*_2_} normal outcomes with the chosen simple linear regression models. Without loss of generality, the intercepts of the two linear equations and means of the two normal predictors were set as *β*_01_ = *β*_02_ = *θ*_1_ = *θ*_2_ = 0. Next, the test statistic T* was computed and the simulated power was the proportion of the 10,000 replicates whose test statistics |T*| exceeded the corresponding critical value $t_{\hat{\nu },0.025}$. Therefore, the adequacy of the two sample size procedures is determined by the error (= estimate power–simulated power) between the estimated power computed from analytic formulas and the simulated power of Monte Carlo study. The simulated power and error are also summarized in Tables 1–6 for all 90 design conditions.

The results in Tables 1–6 indicate that there exists certain discrepancy between the two competing sample size procedures. The outcomes of the simplified *t* method deteriorate to some extent for larger slope differences *β*_1*D*_ and sample size ratios *r*. Correspondingly, the resulting errors in Table 6 with *β*_1*D*_ = 1.00 and *r* = 3 are clearly greater than those in Table 1 with *β*_1*D*_ = 0.50 and *r* = 1 for the identical combination of variance patterns {$\tau_{1}^{2},\;\tau_{2}^{2}$} and $\left\{\sigma_{1}^{2},\sigma_{2}^{2}\right\}$. It is notable that the largest error of the simplified *t* technique is 0.1096 associated with Case 11 in Table 6, whereas the error of Case 11 in Table 1 is only 0.0134. Therefore, the power function Ψ_*ST*_ does not maintain a consistent performance for the conditions examined here and the corresponding sample size computation is especially less accurate for large effect sizes and unbalanced designs. On the other hand, the power discrepancy suggests a close agreement between the estimated power and the simulated power for the MT procedure regardless of the model configurations. Specifically, all the absolute errors of the 90 designs are less than 0.01 and the only exception has an error of 0.0143 occurred with Case 7 in Table 3. Relatively, the error induced by the simplified *t* method is 0.0288 for the same setting. From a practical standpoint of providing a versatile solution without specifically confining to any particular settings, the failure to give reliable performance is obvious limitation of the simplified *t* method. Considering the potentially diverse interaction and predictor configurations of field studies, the mixed *t* approach is relatively more consistent and accurate than the simplified *t* method to be considered as a general tool. In addition, the fundamental behaviors of Type I error control of the two contending power procedures were also examined. Following the sample sizes and other settings in Tables 1–6, simulated Type I errors were computed with 10,000 iterations under the null values *β*_11_ = *β*_12_ = 0. The results show that all the simulated Type I error rates are contained within a close range of the nominal level *α* = 0.05, Specifically, the maximum absolute errors of the simplified *t* and mixed *t* methods are 0.0048 and 0.0056, respectively. Hence, the two contending power approximations of the extended Welch test provide adequate Type I error performance to justify the presented power appraisals for the model configurations considered here.

## Results

### Empirical study

To exemplify the typical situation most frequently encountered in the planning stage of a research study, the numerical data involving the organ weights of crossbred diabetic and normal mice in Bishop [22] is exploited to illustrate the computational aspects of the suggested power and sample size procedures for assessing the interaction effects between a continuous predictor and a dichotomous treatment variable. The example concerns whether the diabetic mice have kidney weights which are disproportionately larger, relative to their body weights, than those of the normal mice.

The subgroup simple regression with body weight (in grams) predicting kidney weight was conducted for the crossed diabetic and normal groups with sample size {*N*_1_, *N*_2_} = {9, 25}. The resulting slope estimates and error variances are $\left\{{\hat{\beta }}_{11},{\hat{\beta }}_{12}\right\}$ = {15.9286, 3.8398} and $\left\{{\hat{\sigma }}_{1}^{2},{\hat{\sigma }}_{2}^{2}\right\}$ = {10124.8980, 9097.9625}, respectively. Moreover, the summary statistics for the body weights are ${\bar{X}}_{1}$ = 42.8889, ${\bar{X}}_{2}$ = 33.8800, ${\hat{\tau }}_{1}^{2}={SSX}_{1}/\kappa_{1}$ = 31.1111, and ${\hat{\tau }}_{2}^{2}={SSX}_{2}/\kappa_{2}$ = 23.8600. The traditional analysis leads to a test statistic *T* = 1.6492 with degrees of freedom *df* = 30 and *p*-value = 0.1098. Alternatively, the computed value of the extended Welch test statistic is T* = 1.6073 with degrees of freedom $\hat{\nu }$ = 12.9349 and *p*-value = 0.1321. Hence, the two tests for a difference between the two slope coefficients is reported as statistically non-significant for *α* = 0.05. In other words, it is concluded that there is no group effect (crossed diabetic and normal groups) on the relationship between the body weight and kidney weight.

### Power and sample size calculations

In view of the advance nature of research design, the practice guidelines suggest that typical sources like published finding or pilot study can give creditable and sensible planning values for the model configurations. The reader is referred to the excellent texts of Cohen [23], Ryan [24], and Shao, Wang, and Chow [25] for essential issues and further details on power analysis and sample size determination. Therefore, the prescribed data of Bishop [22] is employed to provide planning values of the regression parameters, error components, and predictor configurations to guide future diabetic and body weight interaction studies. With the sample sizes of {*N*_1_, *N*_2_} = {9, 25} and significance level *α* = 0.05, the achieved power can be readily computed with the supplemental programs (Supplements A and C). For example, the programming lines for the SAS user specifications in Supplement A are as follows:

*USER SPECIFICATION PORTION;

*DESIGNATED ALPHA;ALPHA = 0.05;

*SAMPLE SIZES;N1 = 9;N2 = 25;

*SLOPE COEFFICIENTS;BETA11 = 15.9286;BETA12 = 3.8398;

*ERROR VARIANCEQ;SIGSQ1 = 10124.8980;SIGSQ2 = 9097.9625;

*PREDICTOR VARIANCES;TAUSQ1 = 31.1111;TAUSQ2 = 23.8600;

*END OF USER SPECIFICATION PORTION;

Also, the corresponding R statements in Supplement C are specified as

**#USER SPECIFICATION PORTION**

**alpha<-0.05 #DESIGNATED ALPHA**

**n1<-9 #SAMPLE SIZES**

**n2<-25**

**beta11<-15.9286 #SLOPE COEFFICIENTS**

**beta12<-3.8398**

**sigsq1<-10124.8980 #ERROR VARIANCES**

**sigsq2<-9097.9625**

**tausq1<-31.1111 #PREDICTOR VARIANCES**

**tausq2<-23.8600**

**#END OF SPECIFICATION**

The result shows that the achieved power of the particular unbalanced design is Ψ_*MT*_ = 0.3013 which is far less than the fairly common level of 0.80. Therefore, the power calculation suggests that the designated configurations with the prescribed sample size scheme do not warrant a decent chance of detecting the slope difference between two treatment groups. Instead, as an illustration of sample size determination for planning balanced study, the supplemental algorithms (Supplements B and D) show that the sample sizes of {*N*_1_, *N*_2_} = {42, 42} and {56, 56} are required to achieve the nominal power levels of 0.80 and 0.90, respectively. The attained powers of the two sample size schemes are marginally greater than the nominal power level with 0.8025 and 0.9050, respectively. As in the previous case of power calculation, the chosen key configurations are included in the user specifications of the SAS/IML and R programs presented in the supplemental files B and D. In supplement B, the required inputs are:

*USER SPECIFICATION PORTION;

*DESIGNATED ALPHA;ALPHA = 0.05;

*NOMINAL POWER;POWER = 0.80;

*SLOPE COEFFICIENTS;BETA11 = 15.9286;BETA12 = 3.8398;

*ERROR VARIANCEQ;SIGSQ1 = 10124.8980;SIGSQ2 = 9097.9625;

*PREDICTOR VARIANCES;TAUSQ1 = 31.1111;TAUSQ2 = 23.8600;

*SAMPLE SIZE RATIO;RN21 = 1;

*END OF USER SPECIFICATION PORTION;

Also, the necessary specifications of supplement D are as follows:

**#USER SPECIFICATION PORTION**

**alpha<-0.05 #DESIGNATED ALPHA**

**power<-0.80 #NOMINAL POWER**

**beta11<-15.9286 #SLOPE COEFFICIENTS**

**beta12<-3.8398**

**sigsq1<-10124.8980 #ERROR VARIANCES**

**sigsq2<-9097.9625**

**tausq1<-31.1111 #PREDICTOR VARIANCES**

**tausq2<-23.8600**

**rn21<-1 #SAMPLE SIZE RATIO**

**#END OF SPECIFICATION**

Accordingly, users can easily identify the statements containing the exemplifying values in the computer code and then modify the inputs to accommodate their own model specifications.

Moreover, in an actual experiment, the available resources are generally limited and the cost for obtaining a crossbred diabetic mouse is often more expensive than a normal one. Consequently, an unbalanced design may be more efficient and a flexible approach allowing unequal sample size allocation is desired. Using a sample size ratio *r* = *N*_2_/*N*_1_ = 3, the necessary sample sizes to attain the nominal power levels of 0.80 and 0.90 are {*N*_1_, *N*_2_} = {28, 84} and {37, 111}, respectively. The two computed sample sizes of 112 (= 28 + 84) and 148 (= 37 + 111) are (112–34)/34 = 2.29 and (148–34)/34 = 3.35 times more than is employed for the original design, respectively. The prescribed explications demonstrate the usefulness of power and sample size assessments in planning a desirable research design. In contrast, without a proper and thorough evaluation, it may lead to some distorted power performance and unsatisfactory research outcome for the conducted study. Note that these exemplifying configurations are listed in the user specifications of the SAS/IML and R programs in the supplemental files. Following the numerical demonstration, researchers can easily identify the input statements in the computer code and then replace the required values with their own model specifications.

## Conclusions and discussion

Assessing interaction effects of categorical treatment variables or testing the equality of regression slopes is a fundamental procedure in biology, medicine, and other fields of science. The present study focuses on the two-group case because it represents the great majority of interactive studies. In view of the frequent violation of the homogeneity of error variance assumption in applications, the extended Welch approach has been proposed as a vital alternative to the ordinary least squares method for the detection of interaction effect between a dichotomous treatment variable and a continuous predictor. However, the associated power computation and sample size determination for interaction analysis remain unavailable. To alleviate the difficulty in detecting interaction due to insufficient statistical power, this article seeks to contribute to the literature by presenting practical solutions to power and sample size calculations for the extended Welch tests of regression slope difference under variance heterogeneity. Accordingly, two distinct techniques are described to emphasize and account for the stochastic nature of the predictor variables for approximating the general distribution and power behavior of the extended Welch statistic. In addition to the detailed theoretical explications for the inherent relationship and distinct property of the two approaches, numerical investigations are conducted to reveal the relative performance of the contending procedures as a reliable technique to accommodate the predictor features that make interaction research particularly distinctive. The suggested approach has the important advantages over the simplified method in theoretical justification, great accuracy, and wide applicability. Due to the limitation of existing software packages and the practical usefulness of the proposed methodology, computer algorithms are presented to implement the recommended power calculations and sample size determinations.

Within the context of statistical point estimation, unbiasedness is an essential principle in the selection of desirable point estimators. However, the unbiased criterion does not guarantee the chance of obtaining a correct estimate. For example, it is well known that the sample mean is unbiased of the population mean. Although the probability of making a correct estimate is zero for the case of continuous variables, the sample mean was not deprived of its role as a reliable and established estimator. For the problem of sample size determination, some researchers may suggest that it makes no difference to apply a simple formula given the many unknowns in the planning stage of research design. Nevertheless, the underlying principle of research design planning is to conduct advance analysis based on the best knowledge of the model configurations. It is prudent to adopt a feasible procedure that incorporates into power and sample size calculations as much information about the model configurations as possible. Note that hierarchical linear models (Raudenbush & Bryk [26]) have the distinct structure for accommodating the notion of random coefficients in the traditional linear models. Here, the stochastic nature of predictor variables is taken into account for the described power and sample size methodology for interaction research. Essentially, the developed algorithms will yield accurate power appraisal and sample size determination provided that all the required features are suitably specified.

In addition to the relaxed scenario of variance heterogeneity, it should be stressed that the recommended power and sample size approaches also require the independence and normality assumptions. The robust feature clearly depends on the extent of how poorly the underlying response and predictor distributions diverge from the normality setting. However, Poncet et al. [27] and Schmider et al. [28] reinvestigated the robustness of two-sample *t* test and ANOVA *F* test against violations of the normal distribution assumption by Monte Carlo simulations. Their results gave strong support for the robustness of the *t* and *F* tests under application of non-normally distributed data. Although they did not evaluate the Welch method, the updated findings in some sense reinforce the documented robustness for the Welch-type procedures under mild departures from normality assumption. More importantly, Lix and Keselman [29] noted that the Welch-type tests preserve reasonably good Type I error control and compare favorably with the traditional method in terms of statistical power for many types and degrees of non-normality likely to be encountered in practical research. Moreover, the established class of generalized linear models offers an excellent and distinct method for analyzing non-normal data. Related details and follow-up procedures can be found in McCullagh and Nelder [30] and McCulloch, Searle, and Neuhaus [31].

## Supporting information

S1 File Approximate power functions of the extended Welch test. (DOCX)

S2 File SAS programs. (DOCX)

S3 File R programs. (DOCX)
