# Supplementary material for: Power and sample size calculations for comparison of two regression lines with heterogeneous variances
Source: PLoS One. 2018 Dec 17;13(12):e0207745. doi: 10.1371/journal.pone.0207745 (PMC6296670; doi:10.1371/journal.pone.0207745)
Supplement: S1 File — (DOCX) [file pone.0207745.s001.docx]

**S1 File**

Appendix

Approximate power functions of the extended Welch test

Note that Welch [13] did not address the distribution of *T** under non-null hypothesis and related power issues. However, it can be shown with the same theoretical arguments and analytic derivations in Welch [13] that the statistic *T** has the general approximate distribution for the given values of *SSX*_1_ and *SSX*_2_:

*T**|[*SSX*_1_, *SSX*_2_] *t*(ν, Δ), (A1)

where *t*(ν, Δ) is a noncentral *t* distribution with degrees of freedom ν and noncentrality parameter Δ with

1/ν = {} + {},

and

Δ = .

Assume the predictors {*X*_1_*_j_*, *j* = 1, ..., *N*_1_} and {*X*_2_*_k_*, *k* = 1, ..., *N*_2_} are *iid* random variables with *E*[*X*_1_*_j_*] = θ_1_, *Var*[*X*_1_*_j_*] = τ, and *E*[*X*_2_*_k_*] = θ_2_, *Var*[*X*_1_*_k_*] = τ, respectively. To obtain an unconditional distribution of *T**, a simple and straightforward approach is to replace the two sum of squares *SSX*_1_ and *SSX*_2_ in Δ with the corresponding expected values *E*[*SSX*_1_] = κ_1_τ and *E*[*SSX*_2_] = κ_2_τ, where κ_1_ = *N*_1_ – 1 and κ_2_ = *N*_2_ – 1. Thus, the overall distribution of *T** can be approximated by a noncentral *t* distribution:

*T** *t*(ν*_ST_*, Δ*_ST_*), (A2)

where

1/ν*_ST_* = {} + {},

Δ*_ST_* = ,

*b*_1_ = κ_1_/κ, *b*_2_ = κ_2_/κ, and κ = κ_1_ + κ_2_. According to this easy approximation, it is described as the simplified *t* method. The corresponding power function can be readily expressed as

Ψ*_ST_*(β_1_*_D_*) = *P*{|*t*(ν*_ST_*, Δ*_ST_*)| > *t*}. (A3)

It is well known that Cohen’s [23] *d* is an estimate of the standardized mean difference δ = (μ_1_ – μ_2_)/σ that reflects the difference between two population means divided by their common standard deviation. In this comparison of two regression slopes, however, no similar or recognized effect size measure has been given in the literature due to the complication of unequal variances and sum of squares of the predictor variables. Assume that the two sample size allocation ratios are fixed as *N*_1_/*N_T_* = *q*_1_ and *N*_2_/*N_T_* = *q*_2_ with *q*_1_ + *q*_2_ = 1. Note that *SSX*_1_/*N*_1_ and *SSX*_2_/*N*_2_ converge in probability to τ and τ for large sample sizes *N*_1_ and *N*_2_, respectively. Consequently, * = Δ/*N_T_* converges in probability to δ* where

* = and δ* = . (A4)

Hence, similar to the Cohen’s [23] δ and *d* in the case of standardized mean difference, the prescribed δ* and * provide a potential effect size measure and a convenient estimator for the standardized slope difference.

To explicate the implications of appropriate reliance on the predictor variables, the continuous predictor variables {*X*_1_*_j_*, *j* = 1, ..., *N*_1_} and {*X*_2_*_k_*, *k* = 1, ..., *N*_2_} are assumed to have the independent normal distributions *N*(θ_1_, τ) and *N*(θ_2_, τ), respectively. The normality setting is commonly employed to provide a convenient framework for analytical derivations and numerical investigations in interaction studies, such as Alexander and DeShon [8], DeShon and Alexander [9], Dretzke et al. [10], Overton [11], and Shieh [12]. It follows from standard results that *K*_1_ = *SSX*_1_/τ ~ χ^2^(κ_1_) and *K*_2_ = *SSX*_2_/τ ~ χ^2^(κ_2_). Moreover, *K* = *K*_1_ + *K*_2_ ~ χ^2^(κ), κ = κ_1_ + κ_2_, *B* = *K*_1_/*K* ~ Beta{κ_1_/2, κ_2_/2}, and Beta{*a*, *b*} is a beta distribution with degrees of freedom *a* and *b*. Note that the random variables *K* and *B* are independent.

Under the additional stochastic considerations of *SSX*_1_ and *SSX*_2_ in terms of *K*_1_ and *K*_2_, the *T** statistic has the following two-stage distribution

*T**|[*B*, *K*] *t*(ν*_MT_*, Δ*_MT_*), *K* ~ χ^2^(κ), and *B* ~ Beta{κ_1_/2, κ_2_/2}, (A5)

where

1/ν*_MT_* = {} + {},

Δ*_MT_* = ,

*B*_1_ = *B*, and *B*_2_ = 1 – *B*. The suggested formulation in Equation A5 employs a mixture of *t* distributions and therefore is referred to as the mixed *t* approach. With the two-stage distribution of *T**, the associated power function can be defined as

Ψ*_MT_*(β_1_*_D_*) = *E_B_E_K_*[*P*{|*t*(ν*_MT_*, Δ*_MT_*)| > *t*}], (A6)

where the expectations *E_B_*[·] and *E_K_*[·] are taken with respect to the distributions of *B* and *K*, respectively.

It is essential to note that the power functions Ψ*_ST_* and Ψ*_MT_* depend on the difference between two slope coefficients {β_11_, β_12_}, error variances {σ, σ}, and predictor variances {τ, τ} through Δ*_ST_* and Δ*_MT_*, respectively, but not the mean values of predictors {θ_1_, θ_2_}. More importantly, with the prescribed normality assumptions for the predictor variables, the power function Ψ*_MT_* has the unique conditional distribution on the chi-square distribution and beta distribution. The degrees of freedom and noncentrality parameter ν*_MT_* and Δ*_MT_* of the conditional *t* distribution are functions of the predictor variables. In contrast, the power function Ψ*_ST_* reduces the reliance on the predictor variables to the corresponding variance components. The particular method provides an attractive formulation because of its the conceptual simplicity and computational ease. However, the substitution of constants κ_1_τ and κ_2_τ for the two random variables *SSX*_1_ and *SSX*_2_ in Δ does not preserve the same probability evaluation for *T**. Such modification is vulnerable to numerical justification. Furthermore, it can be shown that the noncentrality terms Δ*_ST_* and Δ*_MT_* are asymptotically equivalent as sample size goes to infinity.
