# Supplementary material for: Power and sample size calculations for comparison of two regression lines with heterogeneous variances
Source: PLoS One. 2018 Dec 17;13(12):e0207745. doi: 10.1371/journal.pone.0207745 (PMC6296670; doi:10.1371/journal.pone.0207745)
Supplement: S2 File — (DOCX) [file pone.0207745.s002.docx]

**S2 File**

Supplement A

SAS/IML program for computing the power for the extended Welch test of regression slope equality

PROC IML;

*USER SPECIFICATION PORTION;

*DESIGNATED ALPHA;ALPHA=0.05;

*SAMPLE SIZES;N1=9;N2=25;

*SLOPE COEFFICIENTS;BETA11=15.9286;BETA12=3.8398;

*ERROR VARIANCEQ;SIGSQ1=10124.8980;SIGSQ2=9097.9625;

*PREDICTOR VARIANCES;TAUSQ1=31.1111;TAUSQ2=23.8600;

*END OF USER SPECIFICATION PORTION;

BETA1D=BETA11-BETA12;STD1=SQRT(SIGSQ1);STD2=SQRT(SIGSQ2);

NUMINT=100;L=NUMINT+1;DD=1E-6;

COEVEC=({1}||REPEAT({4 2},1,NUMINT/2-1)||{4 1})`;

PRINT N1 N2 BETA11 BETA12 BETA1D ALPHA;

PRINT SIGSQ1 SIGSQ2 TAUSQ1 TAUSQ2;

START MTPOWER;

C1=N1-2;C2=N2-2;

DFK1=N1-1;DFK2=N2-1;DFK=DFK1+DFK2;

DFB1=DFK1/2;DFB2=DFK2/2;

BL=DD;BU=1-DD;INTB=(BU-BL)/NUMINT;BVEC=BL+(INTB#(0:NUMINT))`;

WBPDF=(INTB/3)#COEVEC#PDF('BETA',BVEC,DFB1,DFB2);

CL=DD;CU=QUANTILE('CHISQ',1-DD,DFK);

INTC=(CU-CL)/NUMINT;CVEC=CL+(INTC#(0:NUMINT))`;

WCPDF=(INTC/3)#COEVEC#PDF('CHISQ',CVEC,DFK);

QUAN=J(L,1,0);

DO I=1 TO L;

B1=BVEC[I,1];B2=1-B1;

VBT1=SIGSQ1/(B1#TAUSQ1);

VBT2=SIGSQ2/(B2#TAUSQ2);

DFIB=((VBT1/(VBT1+VBT2))##2)/C1+((VBT2/(VBT1+VBT2))##2)/C2;

DFB=1/DFIB;

DELBKVEC=BETA1D/SQRT((VBT1+VBT2)/CVEC);

TCRIT=TINV(1-ALPHA/2,DFB);

QUAN[I,1]=WCPDF`*(CDF('T',-TCRIT,DFB,DELBKVEC)+SDF('T',TCRIT,DFB,DELBKVEC));

END;

MTPOWER=WBPDF`*QUAN;

FINISH;

RUN MTPOWER;PRINT MTPOWER[FORMAT=8.4];

QUIT;

Supplement B

SAS/IML program for computing the sample sizes for the extended Welch test of regression slope equality

PROC IML;

*USER SPECIFICATION PORTION;

*DESIGNATED ALPHA;ALPHA=0.05;

*NOMINAL POWER;POWER=0.80;

*SLOPE COEFFICIENTS;BETA11=15.9286;BETA12=3.8398;

*ERROR VARIANCEQ;SIGSQ1=10124.8980;SIGSQ2=9097.9625;

*PREDICTOR VARIANCES;TAUSQ1=31.1111;TAUSQ2=23.8600;

*SAMPLE SIZE RATIO;RN21=1;

*END OF USER SPECIFICATION PORTION;

BETA1D=BETA11-BETA12;STD1=SQRT(SIGSQ1);STD2=SQRT(SIGSQ2);

NUMINT=100;L=NUMINT+1;DD=1E-6;

COEVEC=({1}||REPEAT({4 2},1,NUMINT/2-1)||{4 1})`;

PRINT BETA11 BETA12 BETA1D ALPHA POWER;

PRINT SIGSQ1 SIGSQ2 TAUSQ1 TAUSQ2;

START STPOWER;

C1=N1-2;C2=N2-2;

DFK1=N1-1;DFK2=N2-1;

VKT1=SIGSQ1/(DFK1#TAUSQ1);

VKT2=SIGSQ2/(DFK2#TAUSQ2);

DFIA=((VKT1/(VKT1+VKT2))##2)/C1+((VKT2/(VKT1+VKT2))##2)/C2;

DFA=1/DFIA;

DELA=BETA1D/SQRT(VKT1+VKT2);

TCRIT=TINV(1-ALPHA/2,DFA);

STPOWER=CDF('T',-TCRIT,DFA,DELA)+SDF('T',TCRIT,DFA,DELA);

FINISH;

START MTPOWER;

C1=N1-2;C2=N2-2;

DFK1=N1-1;DFK2=N2-1;DFK=DFK1+DFK2;

DFB1=DFK1/2;DFB2=DFK2/2;

BL=DD;BU=1-DD;INTB=(BU-BL)/NUMINT;

BVEC=BL+(INTB#(0:NUMINT))`;

WBPDF=(INTB/3)#COEVEC#PDF('BETA',BVEC,DFB1,DFB2);

CL=DD;CU=QUANTILE('CHISQ',1-DD,DFK);

INTC=(CU-CL)/NUMINT;CVEC=CL+(INTC#(0:NUMINT))`;

WCPDF=(INTC/3)#COEVEC#PDF('CHISQ',CVEC,DFK);

QUAN=J(L,1,0);

DO I=1 TO L;

B1=BVEC[I,1];B2=1-B1;

VBT1=SIGSQ1/(B1#TAUSQ1);

VBT2=SIGSQ2/(B2#TAUSQ2);

DFIB=((VBT1/(VBT1+VBT2))##2)/C1+((VBT2/(VBT1+VBT2))##2)/C2;

DFB=1/DFIB;

DELBKVEC=BETA1D/SQRT((VBT1+VBT2)/CVEC);

TCRIT=TINV(1-ALPHA/2,DFB);

QUAN[I,1]=WCPDF`*(CDF('T',-TCRIT,DFB,DELBKVEC)+SDF('T',TCRIT,DFB,DELBKVEC));

END;

MTPOWER=WBPDF`*QUAN;

FINISH;

N1=5;LOOP=0;

DO UNTIL (STPOWER>POWER | LOOP>1000);

N1=N1+1;N2=N1#RN21;LOOP=LOOP+1;

RUN STPOWER;END;NA1=N1;NA2=NA1#RN21;

N1=MAX(NA1-2,5);LOOP=0;

DO UNTIL (MTPOWER>POWER | LOOP>1000);

N1=N1+1;N2=N1#RN21;LOOP=LOOP+1;

RUN MTPOWER;END;

PRINT N1 N2 MTPOWER[FORMAT=8.4];

QUIT;
