# Supplementary material for: Power and sample size calculations for comparison of two regression lines with heterogeneous variances
Source: PLoS One. 2018 Dec 17;13(12):e0207745. doi: 10.1371/journal.pone.0207745 (PMC6296670; doi:10.1371/journal.pone.0207745)
Supplement: S3 File — (DOCX) [file pone.0207745.s003.docx]

**S3 File**

Supplement C

R program for computing the power for the extended Welch test of regression slope equality

function () {

#USER SPECIFICATION PORTION

alpha<-0.05 #DESIGNATED ALPHA

n1<-9 #SAMPLE SIZES

n2<-25

beta11<-15.9286 #SLOPE COEFFICIENTS

beta12<-3.8398

sigsq1<-10124.8980 #ERROR VARIANCES

sigsq2<-9097.9625

tausq1<-31.1111 #PREDICTOR VARIANCES

tausq2<-23.8600

#END OF SPECIFICATION

beta1d<-beta11-beta12

std1<-sqrt(sigsq1)

std2<-sqrt(sigsq2)

numint<-100

l<-numint+1

dd<-1e-6

coevec<-c(1,rep(c(4,2),numint/2-1),4,1)

mtpowerf<-function(){

c1<-n1-2

c2<-n2-2

dfk1<-n1-1

dfk2<-n2-1

dfk<-dfk1+dfk2

dfb1<-dfk1/2

dfb2<-dfk2/2

bl<-dd

bu<-1-dd

intb<-(bu-bl)/numint

bvec<-bl+intb*(0:numint)

wbpdf<-(intb/3)*coevec*dbeta(bvec,dfb1,dfb2)

cl<-dd

cu<-qchisq(1-dd,dfk)

intc<-(cu-cl)/numint

cvec<-cl+intc*(0:numint)

wcpdf<-(intc/3)*coevec*dchisq(cvec,dfk)

quan<-rep(0,l)

for (i in seq(l)) {

b1<-bvec[i]

b2<-1-b1

vbt1<-sigsq1/(b1*tausq1)

vbt2<-sigsq2/(b2*tausq2)

dfib<-((vbt1/(vbt1+vbt2))^2)/c1+((vbt2/(vbt1+vbt2))^2)/c2

dfb<-1/dfib

delbkvec<-beta1d/sqrt((vbt1+vbt2)/cvec)

tcrit<-qt(1-alpha/2,dfb)

quan[i]<-sum(wcpdf*(pt(-tcrit,dfb,delbkvec)+

pt(tcrit,dfb,delbkvec,lower.tail=FALSE)))

}

mtpower<-sum(wbpdf*quan)

}

mtpower<-mtpowerf()

print("n1,n2,beta11,beta12,beta1d,alpha")

print(c(n1,n2,beta11,beta12,beta1d,alpha))

print("sigsq1,sigsq2,tausq1,tausq2")

print(c(sigsq1,sigsq2,tausq1,tausq2))

print("mtpower")

print(mtpower)

}

Supplement D

R program for computing the sample sizes for the extended Welch test of regression slope equality

function () {

#USER SPECIFICATION PORTION

alpha<-0.05 #DESIGNATED ALPHA

power<-0.80 #NOMINAL POWER

beta11<-15.9286 #SLOPE COEFFICIENTS

beta12<-3.8398

sigsq1<-10124.8980 #ERROR VARIANCES

sigsq2<-9097.9625

tausq1<-31.1111 #PREDICTOR VARIANCES

tausq2<-23.8600

rn21<-1 #SAMPLE SIZE RATIO

#END OF SPECIFICATION

beta1d<-beta11-beta12

std1<-sqrt(sigsq1)

std2<-sqrt(sigsq2)

numint<-100

l<-numint+1

dd<-1e-6

coevec<-c(1,rep(c(4,2),numint/2-1),4,1)

stpowerf<-function(){

c1<-n1-2

c2<-n2-2

dfk1<-n1-1

dfk2<-n2-1

dfk<-dfk1+dfk2

vkt1<-sigsq1/(dfk1*tausq1)

vkt2<-sigsq2/(dfk2*tausq2)

dfia<-((vkt1/(vkt1+vkt2))^2)/c1+((vkt2/(vkt1+vkt2))^2)/c2

dfa<-1/dfia

dela<-beta1d/sqrt(vkt1+vkt2)

tcrit<-qt(1-alpha/2,dfa)

stpower<-pt(-tcrit,dfa,dela)+pt(tcrit,dfa,dela,lower.tail=FALSE)

}

mtpowerf<-function(){

c1<-n1-2

c2<-n2-2

dfk1<-n1-1

dfk2<-n2-1

dfk<-dfk1+dfk2

dfb1<-dfk1/2

dfb2<-dfk2/2

bl<-dd

bu<-1-dd

intb<-(bu-bl)/numint

bvec<-bl+intb*(0:numint)

wbpdf<-(intb/3)*coevec*dbeta(bvec,dfb1,dfb2)

cl<-dd

cu<-qchisq(1-dd,dfk)

intc<-(cu-cl)/numint

cvec<-cl+intc*(0:numint)

wcpdf<-(intc/3)*coevec*dchisq(cvec,dfk)

quan<-rep(0,l)

for (i in seq(l)) {

b1<-bvec[i]

b2<-1-b1

vbt1<-sigsq1/(b1*tausq1)

vbt2<-sigsq2/(b2*tausq2)

dfib<-((vbt1/(vbt1+vbt2))^2)/c1+((vbt2/(vbt1+vbt2))^2)/c2

dfb<-1/dfib

delbkvec<-beta1d/sqrt((vbt1+vbt2)/cvec)

tcrit<-qt(1-alpha/2,dfb)

quan[i]<-sum(wcpdf*(pt(-tcrit,dfb,delbkvec)+

pt(tcrit,dfb,delbkvec,lower.tail=FALSE)))

}

mtpower<-sum(wbpdf*quan)

}

n1<-5

loop<-0

stpower<-0

while(stpower<power & loop<1000){

n1<-n1+1

n2<-n1*rn21

loop<-loop+1

stpower<-stpowerf()

}

n1a<-n1

n1<-max(n1a-2,5)

loop<-0

mtpower<-0

while(mtpower<power & loop<1000){

n1<-n1+1

n2<-n1*rn21

loop<-loop+1

mtpower<-mtpowerf()

}

print("beta11,beta12,beta1d,alpha,power")

print(c(n1,n2,beta11,beta12,beta1d,alpha,power))

print("sigsq1,sigsq2,tausq1,tausq2")

print(c(sigsq1,sigsq2,tausq1,tausq2))

print("n1,n2,mtpower")

print(c(n1,n2,mtpower))

}
